# Spinel–rock salt transformation in LiCoMnO_4−*δ*_

**DOI:** 10.1098/rspa.2014.0991

**Published:** 2016-01

**Authors:** Nik Reeves-McLaren, Joanne Sharp, Héctor Beltrán-Mir, W. Mark Rainforth, Anthony R. West

**Affiliations:** 1 Department of Materials Science and Engineering, University of Sheffield, Sheffield S1 3JD, UK; 2 Departamento de Química Inorgánica y Orgánica, Universidad Jaume I, Avda. Sos Baynat s/n, Castellón 12071, Spain

**Keywords:** LiCoMnO_4_, Rietveld, electron energy loss spectroscopy, X-ray absorption near edge structure, Raman, oxygen loss

## Abstract

The transformation on heating LiCoMnO_4_, with a spinel structure, to LiCoMnO_3_, with a cation-disordered rock salt structure, accompanied by loss of 25% of the oxygen, has been followed using a combination of diffraction, microscopy and spectroscopy techniques. The transformation does not proceed by a topotactic mechanism, even though the spinel and rock salt phases have a similar, cubic close-packed oxygen sublattice. Instead, the transformation passes through two stages involving, first, precipitation of Li_2_MnO_3_, leaving behind a Li-deficient, Co-rich non-stoichiometric spinel and, second, rehomogenization of the two-phase assemblage, accompanied by additional oxygen loss, to give the homogeneous rock salt final product; a combination of electron energy loss spectroscopy and X-ray absorption near edge structure analyses showed oxidation states of Co^2+^ and Mn^3+^ in LiCoMnO_3_. Subsolidus phase diagram determination of the Li_2_O-CoO_*x*_-MnO_*y*_ system has established the compositional extent of spinel solid solutions at approximately 500°C.

## 1. Introduction

**Figure 1.**
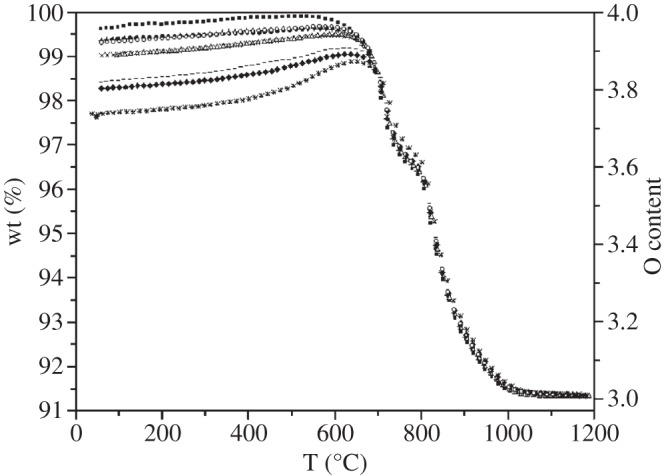
Thermogravimetric data for LiCoMnO_4−*δ*_ samples, from [8].

LiCoMnO_4_ is of considerable interest as a five-volt cathode in lithium ion batteries [1–7]. However, samples are often oxygen-deficient and this introduces an additional, lower voltage redox reaction associated with Li deintercalation and subsequent reintercalation, thereby affecting the performance of the material as a five-volt cathode. LiCoMnO_4_ loses oxygen readily on heating: thermogravimetric, TG, analysis shows loss of up to 25% of its oxygen content over the temperature range approximately 500–1050°C on heating in air (figure 1) [8]. This oxygen loss is accompanied by a transformation from the spinel structure of LiCoMnO_4_ to a cation-disordered rock salt structure of stoichiometry LiCoMnO_3_. Our previous work [9] has shown that this weight loss is reversible on cooling, with the degree of oxygen uptake dependent on the cooling rate; even with relatively fast cooling rates of up to 10°C min^−1^, a single-phase spinel was observed in diffraction data.

Both spinel and rock salt structures have a cubic close-packed oxide ion arrangement with cations distributed over various octahedral and tetrahedral sites. In spinels, the distribution of cations over interstitial sites is often described using the cation inversion parameter, *γ*:

$$\begin{array}{llll} & \gamma =0: & {\left[\text{A}\right]}^{\mathrm{tet}}{\left[{\text{B}}_{2}\right]}^{\mathrm{oct}}{\text{X}}_{4} & =`{\text{normal}}^{\prime }\\ & \gamma =1: & {\left[\text{B}\right]}^{\mathrm{tet}}{\left[\text{AB}\right]}^{\mathrm{oct}}{\text{X}}_{4} & =`{\text{inverse}}^{\prime }\\ & \gamma =0.67: & {\left[{\text{B}}_{0.67}{\text{A}}_{0.33}\right]}^{\mathrm{tet}}{\left[{\text{A}}_{0.67}{\text{B}}_{1.33}\right]}^{\mathrm{oct}}{\text{X}}_{4} & =`{\text{random}}^{\prime }. \end{array}$$

An intriguing question concerns the mechanism by which LiCoMnO_4_, a close-packed oxide, is able to lose 25% of its oxygens on heating and subsequently reincorporate them on cooling: it seems inconceivable that this could take place as a topotactic reaction in which the integrity of the spinel and rock salt lattices is retained throughout.

The main purpose of this paper is to investigate the spinel to rock salt transformation on heating. To do this, samples were quenched from different temperatures and analysed using a complementary range of diffraction, microscopic and spectroscopic techniques. Since it became apparent that the cationic compositions of the spinel and/or rock salt phases were variable during the phase transformation, a subsolidus phase diagram study was also required to determine the possible stoichiometry ranges of the various phases. In completing this study, our interest has been, first, to determine the range of compositions that form a cubic spinel structure under normal conditions of solid-state reaction and, second, to understand the products of decomposition of LiCoMnO_4_ at various temperatures.

Two reports [1,10] have been made previously on the phase diagram of this system, using milligram-scale specimens made via solution-based combinatorial synthesis. Samples were heated at 800°C or 900°C for 3 h and either quenched by removing from the furnace and placing on a metal slab or slow-cooled at approximately 10°C min^−1^ by switching off the furnace.

## 2. Experimental set-up

For initial phase diagram studies, samples were prepared using Li_2_CO_3_ (99+%, dried at 180°C), Co(NO_3_)_2_.6H_2_O (98+%) and (CH_3_CO_2_)_2_Mn.4H_2_O (99+%) as starting reagents (all Sigma-Aldrich). Appropriate weighed amounts were mixed with acetone in an agate mortar and pestle, dried, placed into gold boats, fired at 650°C for decarbonation and at 800°C to complete the reaction. Post-annealing was then conducted to maximize the oxygen contents by holding the specimens at 500°C for 3 days.

For subsequent work to prepare single-phase samples of LiCoMnO_4_ for phase transformation studies, a sol–gel synthesis route was devised using LiNO_3_ (99%), CoCl_2_ (99%) and MnCl_2_ (98%), all Strem Chemicals (Bischeim, France). First, MnCl_2_ was dissolved in ethanol (EtOH) and acetylacetone (acacH) at room temperature. The Mn:EtOH:acacH molar ratio was 1:20:6. If MnCl_2_ was dissolved in ethanol alone, a white suspension was observed after addition of ethanol; this precipitation was avoided by adding acetylacetone. Acetylacetone (acacH) is a rather strong chelating ligand that has often been reported in the sol–gel literature to stabilize non-silicate metal alkoxide precursors. Complexing ligands such as acacH lead to less-hydrolysable M-acac bonds [11]. The resulting solution was stirred for 5 min and then CoCl_2_ and LiNO_3_ were added in sequence. The final mixture was stirred for 4 h and heated under reflux (85°C) for 4 days. The resulting sol led to a transparent gel after evaporation of the solvent, which became a powder after drying under an IR lamp. This powder was heated slowly to 700°C under flowing O_2_, and maintained at 700°C for 48 h with intermittent regrinding using an agate mortar and pestle.

For phase transformation studies, small quantities (approx. 1 g) of this reacted powder were wrapped in Au or Pt foil, heated isothermally in a vertical tube furnace at various temperatures for 1 h, then quenched into liquid nitrogen (200°C s^−1^) in order to preserve high-temperature phase assemblage(s) while preventing re-uptake of oxygen during cooling. These quenched specimens are referred to as Q597, Q679, Q700, Q744, Q849, Q950 and Q1048; the numbers refer to the quench temperature in degree celsius.

X-ray powder diffraction (XRD) used a STOE STADI P diffractometer, Mo K*α*_1_ radiation (λ=0.70930 Å) with a linear position-sensitive detector for lattice parameter measurements and structure refinement. Initial data analysis used the STOE WinX^POW^ software package. Rietveld refinement used the EXPGUI [12] interface for GSAS [13]; the errors quoted are as given by GSAS. Initial isotropic thermal parameters, *U*_iso_, were 0.025 Å^2^ for all positions. The total occupancy of sites was set to unity. The background and scale factors were refined first, using a shifted Chebyschev function with six terms for the background, followed by the lattice and profile parameters. Atomic positions were refined in order of scattering power. The whole process was repeated to convergence with negligible shifts in atomic variables.

Micro-X-ray absorption spectroscopy was conducted on the as-prepared material and Q679, Q700, Q744, Q849, Q950 and Q1048 at the microfocus spectroscopy beamline (I18) at the Diamond Light Source, Didcot, UK. The beam size was around 3×3 μm. Specimens were prepared by dispersion of powders in isopropanol and mounted on lacey carbon films. Elemental mapping was collected over the Co and Mn *K*-edges to check sample homogeneity, before individual spectra were collected over both *K*-edges. A 9-element Ge detector was used. Data treatment used the Athena [14] software package. Single-element distribution maps were prepared using PyMca v. 4.6.2 [15].

Raman spectra were excited with the 514.5 nm line of an Ar laser and recorded in back scattering geometry using a Renishaw inVia micro-Raman spectrometer. Laser power of approximately 2 mW was focused on an approximate 1 μm spot. The spectrometer was equipped with a Peltier-cooled multichannel CCD detector and 2400 l mm^−1^ diffraction grating with slit opening 65 μm and spectral resolution approximately 1 cm^−1^. Data analysis used Igor Pro v. 6.06 [16].

Transmission electron microscopy (TEM) samples were made from as-prepared powder and samples quenched from each temperature by dispersion in isopropanol and collection on a lacey carbon film. Specimens were examined in a JEOL 2010F TEM at 200 kV using electron energy loss spectroscopy (EELS) with a beam size of approximately 15 nm (EDS mode). Spectra were recorded from a number of locations with thickness 0.2–0.8 inelastic mean free paths, determined using the low-loss spectrum. Data were taken from O-K (532 eV), Mn-*L*_2,3_ (640 eV) and Co-*L*_2,3_ (779 eV) edges.

EELS spectra were background-subtracted using a power-law model, and plural scattering deconvoluted using the Fourier-ratio method. Mn valences were determined using the ratio between integrals under the EELS Mn-*L*_3/_/*L*_2_ peaks and background-corrected using the Pearson approach [17]. Reference *L*_3_/*L*_2_ versus valence curves were calculated from spectra taken using MnO_2_ (Mn^4+^), Mn_2_O_3_ (Mn^3+^) and freshly obtained MnO (Mn^2+^). A programme was written using Igor to facilitate this because of the large volume of data collected. Valence determination was also attempted for Co, but was unsuccessful, giving results with error bars so large as to render the results meaningless, so is not included here.

## 3. Results

### (a) Phase diagram studies

**Figure 2.**
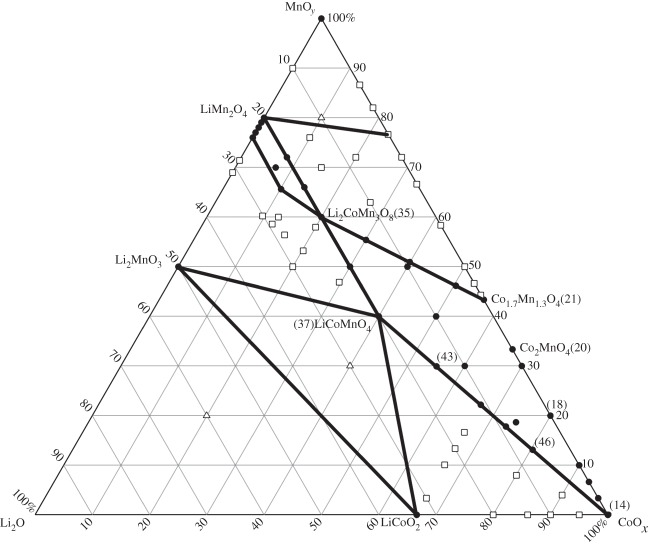
Pseudoternary subsolidus phase diagram at 500°C for the Li_2_O-CoO_*x*_-MnO_*y*_ system; solid lines indicate boundaries of compatibility regions. Selected composition numbers included for reference (see electronic supplementary material, table S1 and table 2). Circles denote single phase, squares, two phases and triangles, three phases.

A number of compositions in the pseudoternary system Li_2_O-CoO_*x*_-MnO_*y*_ system were prepared and by combining these results, electronic supplementary material, table S1, with selected results from the literature, an isothermal section for samples reacted at 800°C in air and given a final annealing at 500°C was obtained and is presented in figure 2. Both Co and Mn can assume a variety of oxidation states, and, therefore, the phase diagram is not truly ternary since it includes different regions in the quaternary system Li-Co-Mn-O. Because of uncertainties in oxygen contents, the results are projected onto the pseudoternary section Li_2_O-CoO_*x*_-MnO_*y*_; this enables the cation contents to be specified but allows variation in the Co/Mn oxidation states.

Samples were prepared by solid-state synthesis with final reaction for 3 days at 800°C to attain thermodynamic equilibrium, followed by post-annealing at 500°C in air to maximize oxygen contents. Reaction at 800°C was chosen since samples heated at lower temperatures, or for less time, often failed to react fully, whereas heating at much higher temperatures risked significant loss of lithia and/or phase transformations associated with loss of oxygen [4,6]. The usual tests for thermodynamic equilibrium were applied, namely that, for a selection of samples, the phase or phase mixture in the products did not change on further heating at the same or slightly higher temperature.

First, we summarize results on the three binary edges and compare these with the literature. Previous reports on spinel formation in Li_2_O-MnO_*y*_ compositions showed the formation of a solid solution between the binary phases LiMn_2_O_4_ and Li_4_Mn_5_O_12_ [18], i.e. Li_1+*x*_Mn_2−*x*_O_4−*δ*_:0≤*x*≤0.33. In our studies, the solubility limit was found to be *x*=0.16 after the final heat treatment at 500°C; this is consistent with the literature since 500°C is above the decomposition temperature of the more Li-rich solid solutions in this series, including Li_4_Mn_5_O_12_.

No evidence was seen for the formation of any spinel phases under our reaction conditions in Li_2_O-CoO_*x*_ compositions, which is also consistent with the literature; previous studies by Levasseur *et al.* [19] found a limited range of Li_1+*x*_Co_1−*x*_O_2_ solid solutions, 0≤*x*≤0.07 formed under oxygen gas at 900°C, though no evidence for this was seen under our synthesis conditions.

A range of cubic spinel solid solutions based on Co_3_O_4_ was found over the composition range Co_3−*y*_Mn_*y*_O_4_: 0<*y*<1.3, which compares reasonably well with that found by Brown *et al.* [10], with a composition limit at *y*=1.08. The minor difference in extent may be due to differences in the heating/cooling regimes used and the difficulty in achieving thermodynamic equilibrium at these temperatures. This is apparent in the high-temperature phase diagram study in air of the system Co-Mn-O by Aukrust & Muan [20], where it is suggested that the compositional extent of the cubic spinel solid solution decreases with decreasing temperature, although no quantitative data were given. Compositions in the range 1.30<*y*<2.16 yielded a mixture of cubic and tetragonal spinels, whose compositions were assumed to be *y*=1.30 and 2.16, respectively, while for *y*≥2.30 two-phase assemblages of tetragonal spinel and bixbyite, Mn_2_O_3_ were obtained.

The pseudoternary phase diagram (figure 2) was constructed using the above results on the binary spinel solid solutions and those given in electronic supplementary material, table S1. It contains two large areas of cubic spinel solid solutions that broadly extend from solid solutions Li_1+*x*_Mn_2−*x*_O_4_ on the Li_2_O-Mn-O edge to the Co_3−*y*_Mn_*y*_O_4_ solid solutions on the Co-Mn-O edge [14,15]. Within these solid solution areas, two ‘ideal’ composition ranges can be identified: first, the join between LiMn_2_O_4_ and LiCoMnO_4,_ with the replacement mechanism, assuming full oxygen stoichiometry, Mn^3+^=Co^3+^ and which also includes the composition, Li_2_CoMn_3_O_8_; second, the join between Co_3_O_4_ and LiCoMnO_4_ with the replacement mechanism, Co^2+^+Co^3+^=Li^+^+Mn^4+^. The two spinel solid solution areas are effectively separated by the composition Li_2_CoMn_3_O_8_, figure 2. One large, quadrilateral-shaped cubic area has compositions containing less than or equal to 20 mole% Li_2_O and is limited by the phase compositions: Co_3_O_4_, Co_1.7_Mn_1.3_O_4_, Li_2_CoMn_3_O_8_ and LiCoMnO_4_. Lattice parameters were refined in GSAS using Le Bail fits (table 1) for a selected number of compositions in this area; a minimum in lattice parameter occurs at LiCoMnO_4_, and unit cell size increases with Co and/or Mn content to a maximum at Co_1.7_Mn_1.3_O_4_.

**Table 1.** Refined lattice parameter for various cubic spinel phases, and for those in LiCoMnO_4_ quenched from various temperatures. Composition number (see electronic supplementary material, table S1) for reference also included.

specimen (composition number)	lattice parameter (Å)
Co_3_O_4_ (14)	8.0838 (19)
Co_2.4_Mn_0.6_O_4_ (18)	8.1808 (10)
Co_2_MnO_4_ (20)	8.2582 (10)
Co_1.7_Mn_1.3_O_4_ (21)	8.3214 (14)
Li_2_CoMn_3_O_8_ (35)	8.1316 (8)
Li_0.78_Co_1.44_Mn_0.78_O_4_ (43)	8.0665 (7)
Li_0.37_Co_2.26_Mn_0.37_O_4_ (46)	8.07629 (17)
as prepared	8.05982 (41)
Q597	8.06705 (34)
Q679	8.07912 (48)
Q700	8.09784 (48)
Q744	8.13072 (61)

The second, smaller area has compositions greater than or equal to 20 mole% Li_2_O and is limited by Li_2_CoMn_3_O_8_ and LiMn_2_O_4_ with a limited range of Li-rich solid solutions whose limit is temperature-dependent. Thus, Robertson *et al.* [21] reported low-temperature synthesis of a complete range of solid solution between Li_4_Mn_5_O_12_ and LiCoMnO_4_ of general formula Li_4−*x*_Mn_5−2*x*_Co_3*x*_O_12_, 0≤*x*≤1 with a maximum synthesis temperature of 440°C, significantly lower than that used in this study. As stated above, at 500°C the LiMn_2_O_4_ solid solutions do not extend as far as Li_4_Mn_5_O_12_ and, therefore, the size of these Li-rich solid solutions with more than 20% Li_2_O is smaller than in the work of Robertson *et al.* [21].

The size and shape of the spinel solid solution areas shown in figure 2 are broadly similar to those reported in [1,10]. Direct comparison is difficult because of the very different synthesis conditions used. In [1,10], small samples were prepared by combinatorial synthesis, initially deposited as liquid mixtures which were dried and fired at 800°C or 900°C for 3 h. Results were very dependent on cooling conditions because oxidation was presumed to occur at slower cooling rates; the results for slow-cooled samples were most similar to those shown in figure 2, but samples were not given a prolonged anneal at 500°C.

The rest of the phase diagram at 500°C is divided into a number of two- and three-phase compatibility regions. Of these, one region bounded by LiMn_2_O_4_, Li_2_CoMn_3_O_8_, Co_1.7_Mn_1.3_O_4_ and approximately Co_0.8_Mn_2.2_O_4_ yields a mixture of two spinel phases, one with cubic and one with tetragonal symmetry. The tetragonal spinel appears to have the composition Co_0.8_Mn_2.2_O_4;_ the cubic spinel is the range of compositions linking Co_1.7_Mn_1.3_O_4_, Li_2_CoMn_3_O_8_ and LiMn_2_O_4_. In the CoO_*x*_-MnO_*y*_ binary system, it has been reported [20] that compositions between Co_1.7_Mn_1.3_O_4_ and Co_0.8_Mn_2.2_O_4_ form a single-phase cubic spinel at elevated temperatures; it may be that Li-containing compositions within the ternary mixed-phase region also follow this behaviour but are beyond the scope of this study.

Samples prepared within the region bounded by LiMn_2_O_4_, MnO_*y*_ and approximately Co_0.8_Mn_2.2_O_4_ yielded a mixture of cubic spinel (LiMn_2_O_4_), tetragonal spinel and bixbyite (Mn_2_O_3_). The tetragonal spinel and bixbyite phases may exist over limited composition ranges, as reported in [1,10].

The join LiCoO_2_–Li_2_MnO_3_ has been extensively studied because of its relevance to lithium battery cathodes. For instance, McCalla *et al.* [1] reported the formation of a complete series of rock salt-related solid solutions in quenched samples, but which were found to phase separate during slow cooling. The endpoints of this coexistence after phase separation were not stoichiometric LiCoO_2_ and Li_2_MnO_3_; instead, LiCoO_2_ contained some Mn and Li_2_MnO_3−*δ*_ contained some Co; similar conclusions were reported in [1]. Consistent with these studies, we note the existence of a three-phase compatibility triangle at 500°C bounded by LiCoO_2_, Li_2_MnO_3_ and LiCoMnO_4_ (figure 2).

### (b) Powder X-ray diffraction studies of LiCoMnO_4−*δ*_

A specimen of LiCoMnO_4_ was synthesized via the sol–gel route using a final heat treatment at 700°C under flowing oxygen as outlined above. It was then slow-cooled to room temperature, in order to maximize oxygen content: the TG data in figure 1 show that oxygen loss in LiCoMnO_4−*δ*_ is continuous with temperature over the range approximately 600–1000°C, but there is also evidence that it occurs in two stages with a poorly resolved intermediate plateau at approximately 775°C.

**Figure 3.**
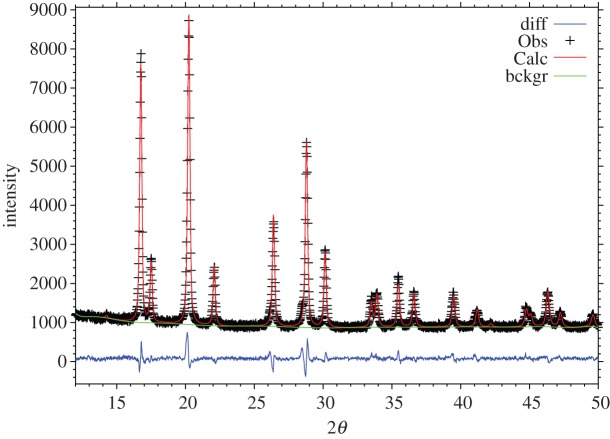
Observed, calculated and difference profiles from XRD data for LiCoMnO_4_ at room temperature. (Online version in colour.)

From powder XRD data, all reflections were indexed using the spinel space group, $\text{Fd}\bar{3}\text{m}$, *a*=8.05982(4) Å, and so, by XRD, the specimen appeared to be single phase. The crystal structure was confirmed using Rietveld refinement to be a normal spinel; Li occupies the 8*a* tetrahedral site, Co and Mn share the 16*d* octahedral site, and oxygen occupies the 32*e* site. After simultaneous refinement of all appropriate parameters, the visual fit of the data (figure 3) was good, as were the quality indicators: *χ*^2^=2.140, *R*_wp_=4.24% and *R*_p_=3.13%. The isotropic thermal parameters were acceptable for all sites; final refined structural parameters are given in table 2.

**Table 2.** Structure refinement parameters and bond lengths for LiCoMnO_4_ at room temperature.

parameter	value
space group	$\text{Fd}\bar{3}\text{m}$
*a* (Å)	8.05982 (41)
*V* (Å^3^)	523.57 (8)
*χ*	2.140
*R*_wp_ (%)	4.24
*R*_p_ (%)	3.13
cation site	8*a*
*x*(=*y*=*z*)	0.125
site occupancy	1.0 Li
*U*_iso_ (Å^2^)	0.0116 (43)
cation site	16*d*
*x*(=*y*=*z*)	0.5
site occupancy	0.5 Co/0.5 Mn
*U*_iso_ (Å^2^)	0.0004 (4)
oxygen site	32*e*
*x* (=*y*=*z*)	0.26145 (26)
site occupancy	1.0 O
*U*_iso_ (Å^2^)	0.0028 (8)
bond lengths (Å)	
Li-O	1.904(1)×4
(Co,Mn)-O	1.927(1)×6

**Figure 4.**
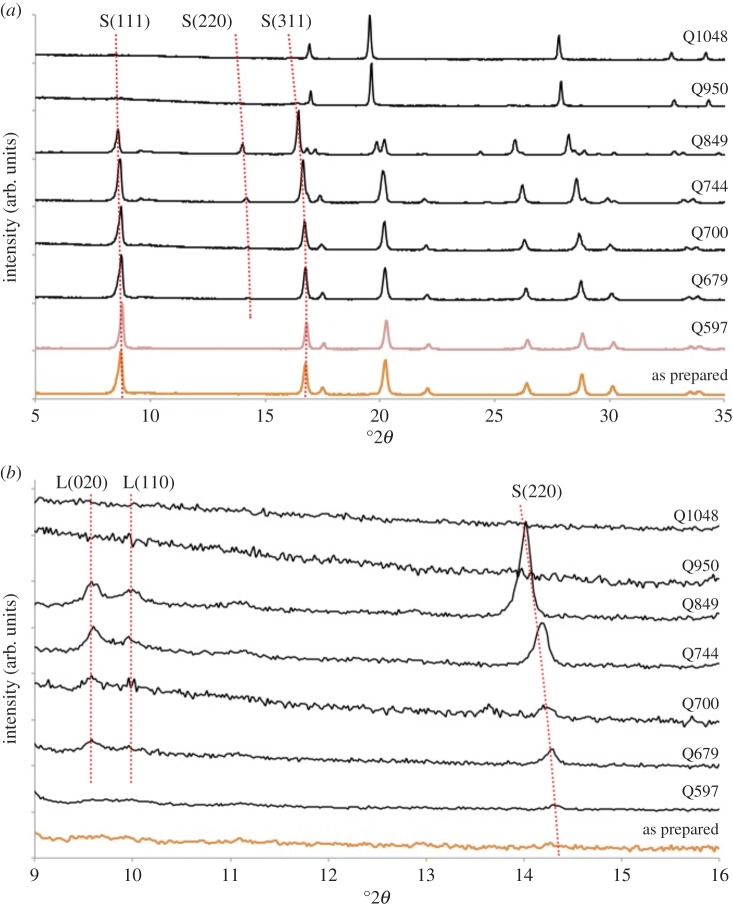
(*a*) X-ray diffraction patterns for as-prepared and quenched specimens of LiCoMnO_4_ and (*b*) expanded view to show Li_2_MnO_3_ peaks, L(110), L(020) and growth of S(220) spinel line with quench temperature. (Online version in colour.)

XRD data collected from quenched samples showed a complex series of changes on heating (figure 4*a*). Phase-pure cubic spinel structure was retained in Q597. Data for Q679 indicated that decomposition had begun with the appearance of Li_2_MnO_3_. Although the main (001) reflection for this layered rock salt overlaps with the spinel (111) reflection at *ca* 8.7°2*θ*, the (020) and (110) reflections from the layered rock salt phase are diagnostic, first appear for Q679 and increase in intensity with quench temperature, as shown at 700°C, 744°C and 849°C (figure 4*b*). In Q849, peaks from a third phase with a disordered rock salt XRD pattern were observed (figure 4*a*). Q950 showed mainly the disordered rock salt phase, with a small spinel peak at approximately 16°2*θ*; Q1048 was phase-pure disordered rock salt. At this temperature, TG data (figure 1) show that 25% of the oxygen content has been lost giving a rock salt stoichiometry ‘LiCoMnO_3_’.

Lattice parameters of the spinel phase in quenched samples (table 1) show a nonlinear increase with quench temperature. There are two possible causes of this, both of which would be consistent with our results. First, oxygen loss may cause a reduction in oxidation state of Co and/or Mn. Second, the spinel phase may become richer in Co, consequent upon precipitation of Li_2_MnO_3_.

As well as the various phase changes observed on heating, changes in certain line intensities of the spinel phase occurred, particularly the (220) peak at approximately 14°2*θ* (figure 4*a*,*b*). For cubic spinels, the (220) line in XRD data is especially sensitive to the occupancy of the 8*a* tetrahedral site. If the spinel is fully normal and Li occupies the 8*a* site, this line should be effectively absent; however, if cation mixing occurs, with a heavier scatterer (e.g. Co and/or Mn) on this site, then the line intensity should increase. Such behaviour is readily apparent in figure 4*b*, suggesting that cation mixing on the 8*a* site occurs during heating.

An analysis of intensity ratios for the spinel (111) and (311) lines provides a further sensitive indicator of cation distribution. In as-prepared LiCoMnO_4_, this ratio was 1.37 compared to 0.12 for Co_3_O_4_ and 0.10 for Co_2_MnO_4_. This ratio remained constant, within reasonable errors, for Q597, Q679 and Q700, but decreased to 1.01 for Q744 and 0.26 for Q849. This decrease is further evidence for increased occupancy of the 8*a* sites by Co/Mn and is consistent with the spinel phase becoming increasingly Li-deficient on heating, as a consequence of decomposition of the original LiCoMnO_4_ into Li_2_MnO_3_- and Co_2_MnO_4_-like phases.

Results on the high-temperature decomposition of LiCoMnO_4_ can be interpreted qualitatively in terms of the phase diagram (figure 2) although the diagram refers to equilibration at 500°C. The appearance of Li_2_MnO_3_ at intermediate temperatures indicates that the spinel becomes Li-deficient and, indeed, may lie on a line passing through Li_2_MnO_3_ and LiCoMnO_4_ and towards, but not extending as far as, Co_2_MnO_4_. This is supported by changes in XRD intensities of the spinel referred to above and is further supported by separate observations that synthesis of phase-pure LiCoMnO_4_ requires a final anneal at low temperatures in order to both optimize oxygen content and prevent formation of Li_2_MnO_3_ [5–7,22].

At the highest temperatures studied, a disordered rock salt phase appears and is phase-pure at 1048°C. From TG results (figure 1) LiCoMnO_4_ has lost up to 25% of its oxygen at these temperatures and therefore the rock salt phase has the overall stoichiometry LiCoMnO_3_. It is well known that under appropriate conditions of low oxygen partial pressure, CoO and MnO have the rock salt structure; both LiCoO_2_ and Li_2_MnO_3_ have ordered rock salt structures but appear not to disorder prior to melting. We may therefore add the composition LiCoMnO_3_ to the list of phases that exhibit a high-temperature rock salt structure and also speculate that a large area(s) of compositions in the pseudoternary system Li_2_O-Mn-Co-O, with appropriate adjustment of their oxygen contents, may exist with the cation-disordered rock salt structure.

### (c) X-ray absorption near edge spectroscopy

**Figure 5.**
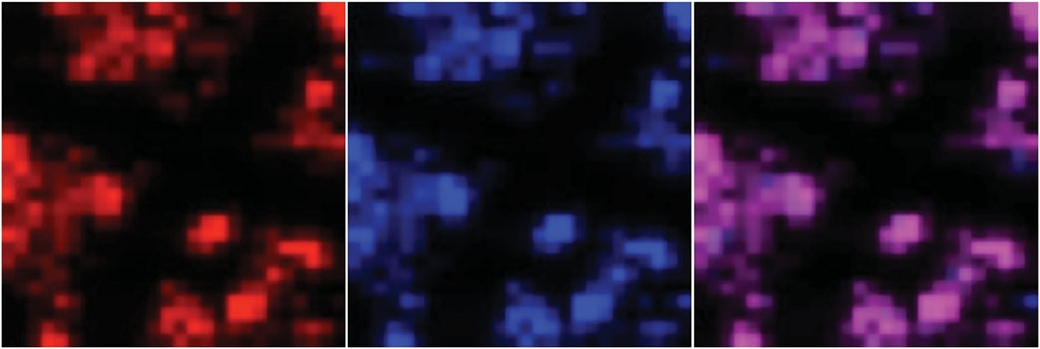
Colour-coded single-element distribution maps for Mn (red), Co (blue) and Mn, Co together, for as-prepared LiCoMnO_4_.

The homogeneity of as-prepared LiCoMnO_4_ was checked initially using maps of X-ray absorption (figure 5) collected at the Mn and Co *K*-edge energy levels in raster fashion across a sample that had an area including multiple grains. Essentially, brighter colours indicate a greater concentration of that element. Each pixel on the maps refers to a 5×5 μm area spot size. On combining Mn (figure 5*a*) and Co (figure 5*b*) maps to form the overall map in figure 5*c*, any areas lacking in either element should be readily apparent; the general lack of red or blue ‘pixels’ on the combined map indicates a reasonable level of metal homogeneity on this length scale.

**Figure 6.**
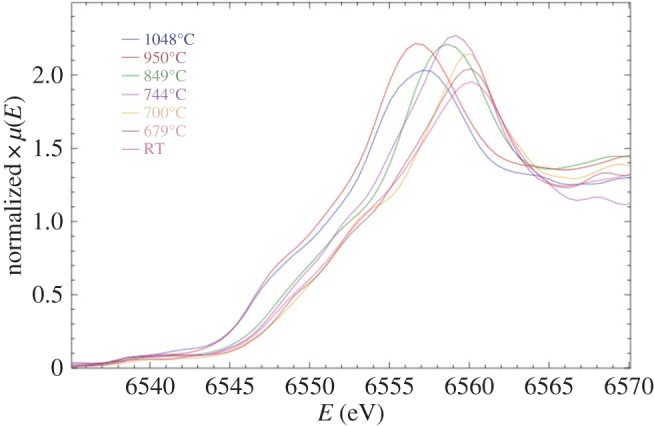
Mn *K*-edge XANES spectra for LiCoMnO_4_ quenched from various temperatures.

**Figure 7.**
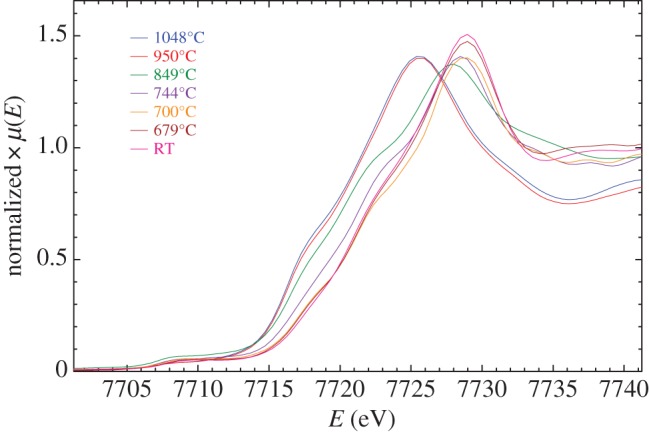
Co *K*-edge XANES spectra for LiCoMnO_4_ quenched from various temperatures.

**Figure 8.**
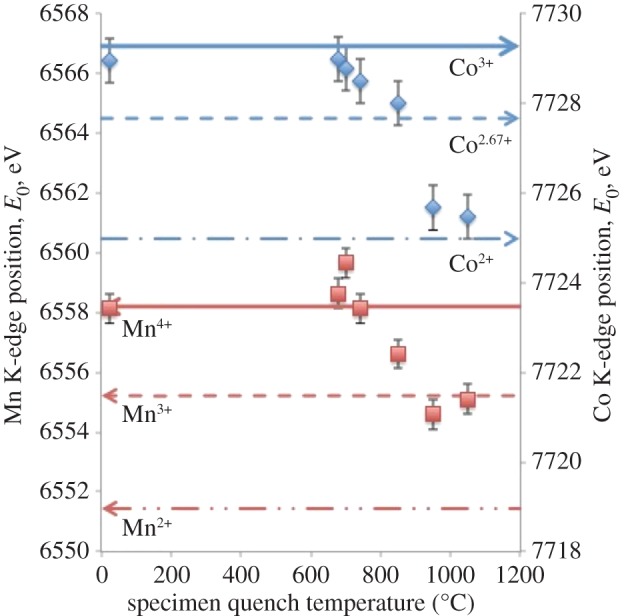
Determination of average Mn and Co valence states from XANES data of as-prepared and quenched LiCoMnO_4_ using calibration peak maximum values measured from standard materials: C_4_H_6_Mn^2+^O_4_.4H_2_O (red dotted dashed line), Mn^3+^_2_O_3_ (red dashes) and Mn^4+^O_2_ (red solid line), Co^2+^O (blue dotted dashed line), Co^2.67+^_3_O_4_ (blue dashes) and LiCo^3+^O_2_ (blue solid line).

Mn and Co K-edge spectra for LiCoMnO_4_ quenched from different temperatures are shown in figures 6 and 7. Standard samples containing known valences of Mn and Co were also measured (not shown). In order to extract valence state information from the spectra of quenched samples, the maxima of their absorption edges were compared with those of the standards, as summarized in figure 8 and table 3. The as-prepared sample and specimens quenched from 679°C, 700°C and 744°C gave Mn K-edge maxima that matched well with that of the Mn^4+^ reference. In samples quenched from higher temperatures, E_0_ shifted to lower energies (figure 6) corresponding to a decrease in average Mn oxidation state, over micrometre length scales. Samples quenched from 950°C and 1048°C gave edge maxima close to that observed for the Mn^3+^ reference (figure 8).

**Table 3.** Mn and Co *K*-edge positions for reference standard materials and as-prepared and quenched LiCoMnO_4_ specimens. Errors in *E*_0_ estimated at the size of one data point, ±0.5 eV.

Mn *K*-edge		Co *K*-edge	
reference materials	*E*_0_ (eV)	reference materials	*E*_0_ (eV)
C_4_H_6_Mn^2+^O_4_.4H_2_O	6551.4	Co^2+^O	7725.0
Mn^3+^_2_O_3_	6555.2	Co^2.67+^_3_O_4_	7727.7
Mn^4+^O_2_	6558.2	LiCo^3+^O_2_	7729.3

LiCoMnO_4−∂_ specimens			
quench temperature (°C)	*E*_0_ (eV)	*E*_0_ (eV)	
as prepared	6558.1	7729.0	
679	6558.6	7729.0	
700	6559.7	7728.8	
744	6558.1	7728.5	
849	6556.6	7728.0	
950	6554.6	7725.7	
1048	6555.1	7725.5	

For the Co *K*-edge spectra, a reasonable match in edge position was found for LiCo^3+^O_2_, the as-prepared specimen and those quenched from 679°C and 700°C. The edge position moved to lower energies with increasing quench temperature (figure 7) corresponding to a reduction in the average oxidation state from Co^3+^ to almost Co^2+^ (table 3 and figure 8).

The interpretation of these XANES data is that in LiCoMnO_4_, Co and Mn are essentially in +3 and +4 oxidation states, respectively. The Co oxidation state might be slightly less than +3 if the LiCoMnO_4_ were slightly oxygen-deficient: the sample was not heated in high pressure oxygen as part of the post-reaction anneal. With the onset of oxygen loss on heating, the Co oxidation state starts to reduce (figure 8) at a lower temperature, 690±30°C, than reduction of the Mn oxidation state, 800±55°C; therefore, the initial oxygen loss appears to be charge-compensated by reduction of the Co valence state. At temperatures where Li_2_MnO_3_ appears as a secondary phase, 744°C and 849°C, the Mn oxidation state starts to reduce; as there is little evidence of significant oxygen loss from Li_2_MnO_3_ [23], it appears that Mn in the spinel phase starts to reduce at 849°C, whereas the Co valence state is already clearly a mixture of 2+ and 3+ at these temperatures. At the highest temperatures, 950°C and 1048°C, where the structures are cation-disordered, phase-pure rock salt, the valence states are Co^2+^ and Mn^3+^, consistent with the general formula LiCo^2+^Mn^3+^O_3_.

### (d) Raman spectroscopy

**Figure 9.**
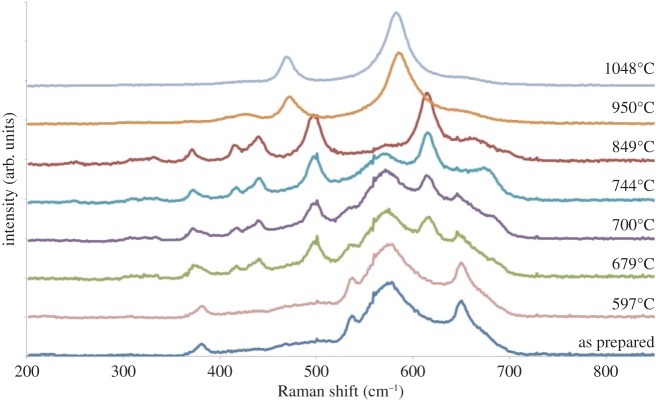
Raman spectra for as-prepared and quenched LiCoMnO_4_. (Online version in colour.)

Raman spectra (figure 9) for as-prepared and quenched LiCoMnO_4_ powders can be divided into three groups:

(i) Spectra of as-prepared powder and that quenched from 597°C are similar to that reported previously by Dokko *et al.* [24] and show four broad lines at 652, 581, 539 and 384 cm^−1^, which can be attributed to A_1*g*_, F^(1)^_2*g*_, F^(2)^_2*g*_ and either E_*g*_ or F^(3)^_2*g*_ modes, respectively [24–28]. An additional line seen by Dokko *et al.* at 692 cm^−1^, and attributed to the presence of Co_3_O_4_ was not observed here.
(ii) Specimens quenched from 679, 700, 744 and 849°C retain these four spectral lines, but also exhibit four new lines at *ca* 619, 501, 445 and 420 cm^−1^ which match those of Li_2_MnO_3_ [29]. The lines of both spinel and layered rock salt phases shift to slightly lower wavenumbers with increasing quench temperature and the intensities of the spinel lines diminish on further heating.
(iii) Specimens quenched from 950 and 1048°C showed only two broad lines, at *ca* 476 and 588 cm^−1^, which we attribute to modes from a disordered rock salt phase, consistent with similar spectra reported for CoO [30,31].

In conclusion, the Raman results agree with those obtained by XRD concerning the sequence of phase changes and the temperatures over which they occur.

### (e) Transmission electron microscopy

**Figure 10.**
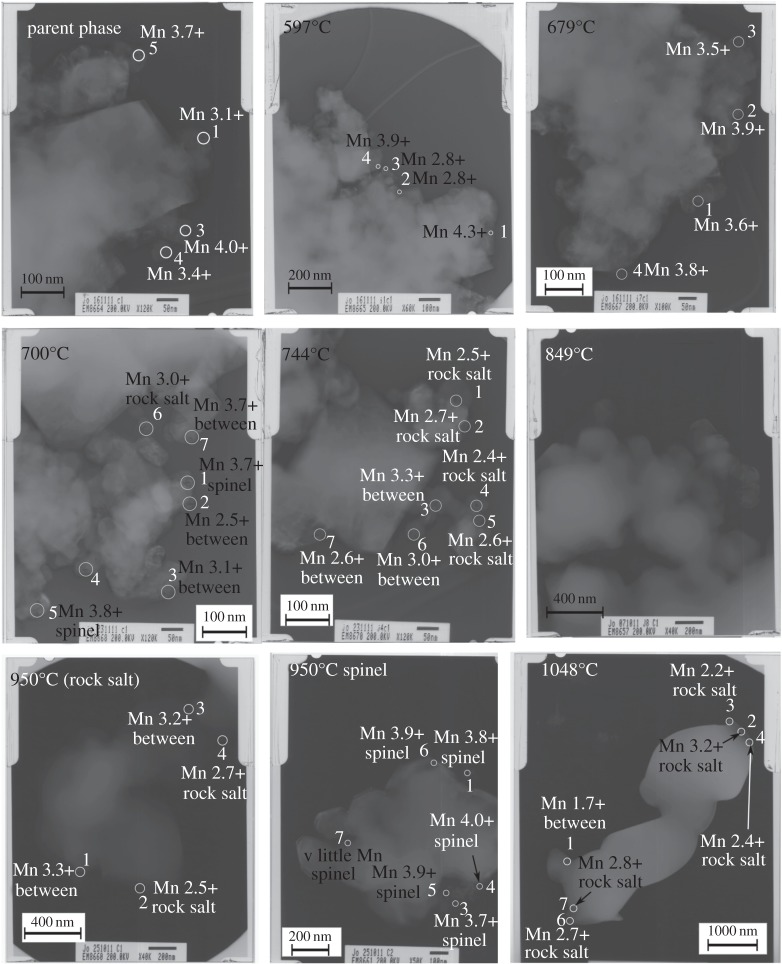
TEM images from quenched samples.

TEM images are shown in figure 10; three broad regimes of particle morphology can be observed. First, in the as-prepared specimen and those quenched from 700°C and below, TEM images showed slightly faceted crystallites with an approximate average diameter of 100 nm. Second, more strongly faceted crystallites were observed in Q744 and, with a roughly fourfold increase in size in Q849.

Third, in Q1048 much larger, rounded particles were observed, with size of approximately 1 μm. For Q950, two distinct populations were observed, with a mixture of both rounded and faceted particles. Preparation of suitably thin specimens for TEM analysis was difficult for specimens quenched from 950°C and 1048°C, as these also showed a tendency to form agglomerates.

### (f) Electron energy loss spectroscopy

EELS analyses were conducted on the Mn-*L*_2,3_. Co-*L*_2,3_ and O-K edges. For each sample, multiple points were analysed across several grains that were thin enough for study.

**Figure 11.**
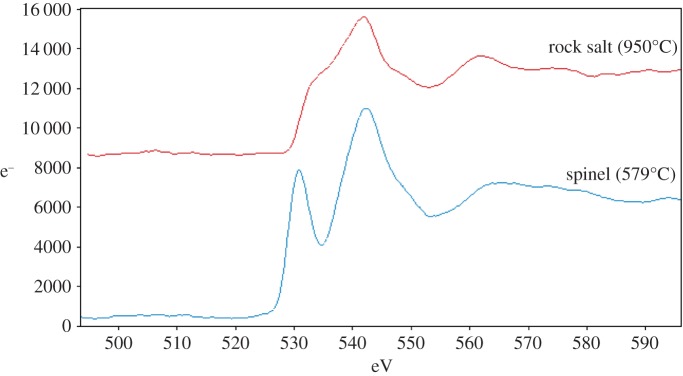
O *K* EELS edges from cubic spinel LiCoMnO_4_ (Q597), and disordered rock salt (Q950). (Online version in colour.)

**Figure 12.**
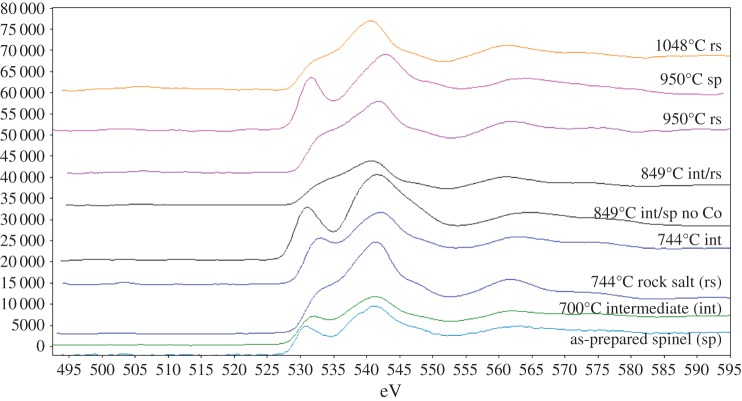
O *K* edges from different quench temperatures, scaled and offset to fit on the same axes. Labelling shows attribution of edge to spinel (sp), rock salt (rs) or intermediate (int) regions. (Online version in colour.)

In the low-temperature parent phase, LiCoMnO_4_, the O-K edge is characteristic of a spinel-type oxygen lattice, whereas in specimens quenched from higher temperatures, O-K edges resemble those of rock salt-type oxygen lattices [32]. Examples of these two extremes are shown in figure 11. Between these extremes, intermediate O-K edge shapes and positions were found; typical examples are given in figure 12. Least-squares fitting of the intermediate O-K edge to a mixture of rock salt and spinel edges yielded results with high *χ*^2^-values. This indicates that the two-phase composite structure is more complex than simple, side-by-side coexistence of discrete spinel and rock salt regions within the illuminated sample volume.

One possibility is that the pre-peak seen in the spinel edge is a sign of oxygen loss caused by beam damage, as reported for some other oxides [33]; in this event, the pre-peak should grow and subsequently shrink, as described in [17], during beam exposure. This possibility was tested, but the intensity of the pre-peak relative to the rest of the O-K edge remained unchanged; instead, the whole edge decreased in intensity on prolonged exposure, after which a hole was observed in the sample. The pre-peak is, therefore, a genuine feature of the sample; perhaps, the lattice itself has an inhomogeneous distribution of O vacancies, for example.

**Figure 13.**
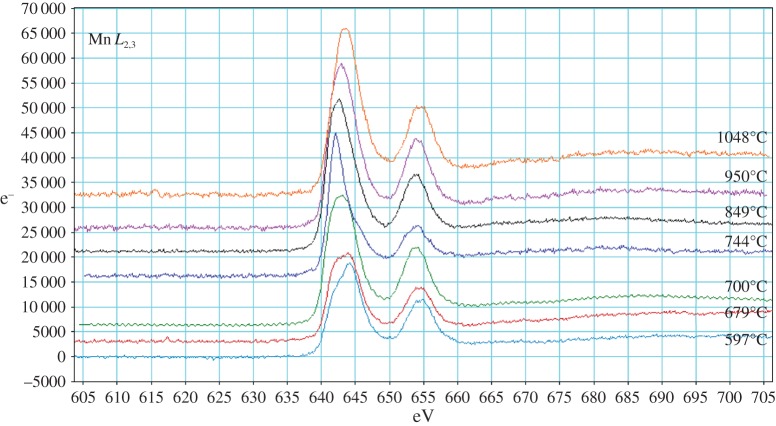
Mn *L*_2,3_ edges from material quenched at different temperatures, offset and scaled to fit on same axes. (Online version in colour.)

**Figure 14.**
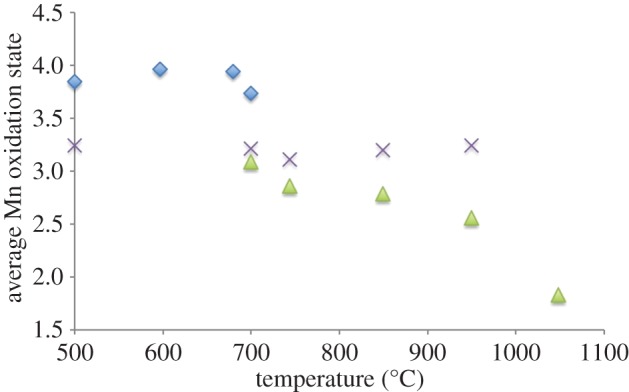
Averaged Mn oxidation state obtained from EELS data differentiated by O *K* edge shape and position (blue diamonds, spinel; purple crosses, Li_2_MnO_3_; green triangles, disordered rock salt). (Online version in colour.)

Mn-*L*_2,3_ edges from each quench temperature are shown in figure 13. Mn oxidation states were derived from *L*_3_/*L*_2_ peak ratio analysis. The derived oxidation states are lower than those found from XANES data, indicating there are likely to be systematic errors in the absolute values. This is probably related to (i) the analysis coming only from the edges of the grains where they were thin enough for EELS analysis and/or (ii) the limited number of standard data points used to construct the Mn oxidation state reference curve, leading to a displacement in the reference curve's position. The shape of this reference curve is less in question, however, so the positions of derived Mn oxidation states relative to each other and, in conjunction with the O-K edge analyses, are instructive. Consequently, the Mn oxidation states and O-K edges from EELS can be assigned to three general regimes (figure 14).

First, spinel-like materials were observed for as-prepared LiCoMnO_4_, Q597, Q679 and Q700. *L*_3_/*L*_2_ peak ratios gave average Mn oxidation states of approximately 3.9+, with the exception of Q700, where an average of Mn^3.73+^ was calculated.

Second, very small inclusions of layered rock-salt Li_2_MnO_3_ were found under most conditions, including in the as-prepared material. In each case, the average Mn oxidation state calculated from *L*_3_/*L*_2_ peak ratios was around 3.2+. The exceptions were Q597 and Q1048. This implies that the parent phase may locally have oxygen-deficient regions and associated Li_2_MnO_3_-type inclusions which were annealed out on heating at approximately 597°C and no longer present at 1048°C.

Finally, O-K edges indicative of rock salt lattices were observed in all specimens quenched from 700°C or higher. The average Mn oxidation state decreased with increasing quench temperature, from 3.1+ in Q700 to 1.8+ in Q1048 (as mentioned previously in this section, absolute values have systematic errors but relative values are reliable).

**Figure 15.**
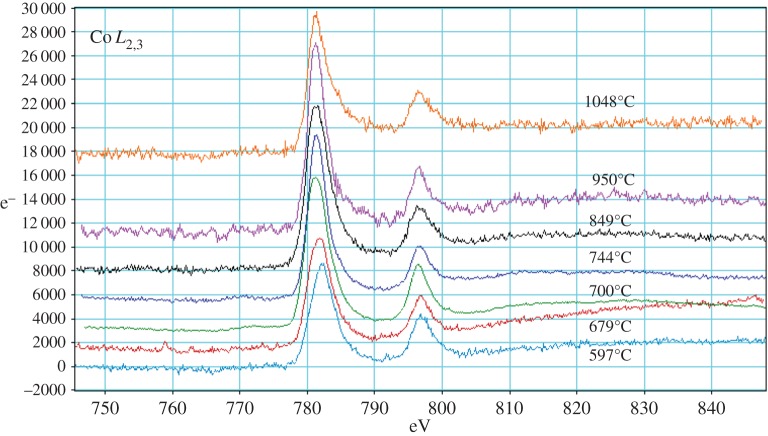
Top: Co *L*_2,3_ edges from different temperatures, offset and scaled to fit on axes. (Online version in colour.)

The observed Co-*L*_2,3_ edges are presented in figure 15. The signal : noise ratio is significantly poorer at this higher energy loss and, therefore, there is more error in the calculated oxidation states. We were unable to observe any statistically significant changes in Co *L*-edge shapes or positions with quench temperature and, as a result, there was minimal sensitivity to the changes in oxidation state expected in these samples. Nevertheless, the presence or the absence of Co *L*-edge peaks could at least be used to analyse local Co distributions in grains of different phases throughout the specimen. Co was found in all three (spinel, layered rock-salt and disordered rock salt) phases in specimens collected from all temperatures, though some particles that did not show a Co edge in EELS analysis, mainly from 849°C, were also observed.

## 4. Discussion

The combination of techniques that probe structure/composition over different length scales gives a good picture of the difficulty in preparing single-phase homogeneous ceramic powders of LiCoMnO_4_, despite (i) the use of sol–gel techniques to promote homogenization of the reaction product and (ii) reaction in an oxygen atmosphere to ensure maximum reaction without oxygen loss.

XRD data on as-prepared specimens showed that single-phase LiCoMnO_4_ was obtained. Data from micrometre-scale probes—Raman spectroscopy and XANES—are in good agreement with the XRD analysis; in particular, no evidence of any secondary phases was found via Raman spectra collected from various spots. However, the nano-scale EELS technique showed inhomogeneities in thinner parts of TEM specimens and regions in which the Mn oxidation state is reduced to below four, possibly due to the loss of oxygen from sample surfaces.

The results obtained from the combination of characterization techniques show that, on heating, weight loss from LiCoMnO_4_ proceeds in essentially three stages:

(i) A drop in oxygen content commences approximately above 600°C, associated primarily with reduction of Co and segregation of Li_2_MnO_3_. It is not known how much oxygen loss can be tolerated by LiCoMnO_4_ prior to precipitation of Li_2_MnO_3_ due to the relative insensitivity of the bulk phase analysis techniques, XRD and Raman spectroscopy, to small amounts of secondary phases, but there is clear evidence of precipitation of Li_2_MnO_3_ by 679°C. From consideration of the phase diagram, it is reasonable to assume that the spinel phase therefore becomes increasingly Li-deficient following the general reaction:
  $${\text{LiCoMnO}}_{4}\to x{\text{Li}}_{2}{\text{MnO}}_{3}+{\text{Li}}_{1-2x}{\text{CoMn}}_{1-x}{\text{O}}_{4-3x-\delta }+\delta /2{\text{O}}_{2}$$
(ii) We do not know if the Li_2_MnO_3_ precipitate is stoichiometric, contains a small amount of Co, or is slightly oxygen-deficient.
(iii) A short intermediate weight loss ‘plateau’ occurs at approximately 780°C, where the rate of oxygen loss slows; the overall oxygen content is approximately 3.67. If the lithium-deficient spinel phase has retained a cation : oxygen ratio of 3 : 4, then *δ*=*x*, giving *x*≈0.33. The spinel composition at 780°C would, therefore, be close to Li_0.33_CoMn_0.67_O_2.67_ (i.e. Li_0.5_Co_1.5_MnO_4_ or Li(Co_3_Mn_2_)O_8_), which is similar to other known spinel phases such as LiFe_5_O_8_ and LiAl_5_O_8_.
(iv) Oxygen loss continues on further heating above 800°C, as the intermediate phases recombine to yield a single-phase, disordered rock salt structure by approximately 1000°C. Thus, the intermediate spinel phase, LiCo_3_Mn_2_O_8_, loses more oxygen and cation homogenization occurs with the overall reaction:
  $${\text{Li}}_{0.33}{\text{CoMn}}_{0.67}{\text{O}}_{2.67}+0.33{\text{Li}}_{2}{\text{MnO}}_{3}\to {\text{LiCoMnO}}_{3}+1/3{\text{O}}_{2}$$

The driving force for oxygen loss on heating LiCoMnO_4_ may, therefore, be the precipitation of Li_2_MnO_3_ which, compared to spinel, is effectively oxygen-deficient. It may be that significant oxygen deficiency does not exist in LiCoMnO_4_ as previously suggested, but rather that LiCoMnO_4_ simply has an upper limit of stability, and on heating above 600°C it shows retrograde solubility to give a precipitate of Li_2_MnO_3_ and a non-stoichiometric, Li-deficient spinel.

## 5. Conclusion

The fundamental question concerning the mechanism by which LiCoMnO_4_ spinel transforms to LiCoMnO_3_ with a cation-disorded rock salt structure has been resolved. It is not a topotactic reaction involving reversible loss and subsequent uptake of 25% of the oxide ions from the cubic close-packed oxygen sublattice. Instead, in the first stage, LiCoMnO_4_ becomes a supersaturated solid solution on heating and precipitates Li_2_MnO_3_ together with associated oxygen loss. At the same time, the spinel host structure becomes Li-deficient, with an increased Co : Mn ratio and a composition that approximates to Li_0.5_Co_1.5_MnO_4_.

In the second stage, Li_2_MnO_3_ and Li_0.5_Co_1.5_MnO_4_ re-react, with further oxygen loss from the spinel component, to give a cation-disordered rock salt phase, LiCoMnO_3_. Hence, the final product, LiCoMnO_3_, has the same cation composition as the LiCoMnO_4_ starting phase but with 25% less oxygen; the transformation passes through an intermediate two-phase equilibrium assemblage in which the cationic compositions of the two phases are very different and probably temperature-dependent. The overall mechanism therefore involves cation segregation and subsequent rehomogenization at the same time as oxygen loss occurs. TEM studies show significant changes in particle size and shape, indicating nucleation and growth mechanisms for the different stages. A homogeneous topotactic phase transformation does not occur.

## Supplementary Material

Supplementary Table 1
