# Supplementary material for: Spinel–rock salt transformation in LiCoMnO4−δ
Source: Proc Math Phys Eng Sci. 2016 Jan;472(2185):20140991. doi: 10.1098/rspa.2014.0991 (PMC4786028; doi:10.1098/rspa.2014.0991)
Supplement: Supplementary Table 1 [file rspa20140991supp1.docx]

Supplementary Table 1. Results of heat treatments on compositions in the Li-Co-Mn oxide pseudoternary phase diagram. All samples were heated at 800 °C for 45 h followed by a final anneal at 500 °C for 72 h in air. Key: C = Co_3_O_4_; M = Mn_2_O_3_; L = Li_2_CO_3_; LC_2_ = LiCoO_2_; LM = Li_2_MnO_3_; CS = Cubic spinel, indexed on Fd-3m; TS = Tetragonal spinel, indexed on I4_1_/amd. *Starting compositions were based on Co^2+^ and Mn^2+^ but oxidation to various extent occurred during the heat treatments.

| Composition Number | mol% Li_2_O | mol% CoO_x_* | mol% MnO_y_* | Phases present |
| --- | --- | --- | --- | --- |
| 1 | 5 | 95 | 0 | LC_2_+C |
| 2 | 10 | 90 | 0 | LC_2_+C |
| 3 | 14 | 86 | 0 | LC_2_+C |
| 4 | 20 | 80 | 0 | LC_2_+C |
| 5 | 10 | 0 | 90 | CS+M |
| 6 | 31 | 0 | 69 | CS+LM |
| 7 | 20 | 0 | 80 | CS (LiMn_2_O_4_) |
| 8 | 20.97 | 0 | 79.03 | CS |
| 9 | 21.95 | 0 | 78.05 | CS |
| 10 | 22.95 | 0 | 77.05 | CS |
| 11 | 23.97 | 0 | 76.03 | CS |
| 12 | 28.57 | 0 | 71.43 | LM+ CS |
| 13 | 33.33 | 0 | 66.67 | LM+ CS |
| 14 | 0 | 100 | 0 | CS |
| 15 | 0 | 96.67 | 3.33 | CS |
| 16 | 0 | 93.33 | 6.67 | CS |
| 17 | 0 | 90 | 10 | CS |
| 18 | 0 | 80 | 20 | CS |
| 19 | 0 | 70 | 30 | CS |
| 20 | 0 | 66.67 | 33.33 | CS |
| 21 | 0 | 56.67 | 43.33 | CS |
| 22 | 0 | 55.67 | 44.33 | CS+TS |
| 23 | 0 | 53.33 | 46.67 | CS+TS |
| 24 | 0 | 50 | 50 | CS+TS |
| 25 | 0 | 41.67 | 58.33 | CS+TS |
| 26 | 0 | 33.33 | 66.67 | CS+TS |
| 27 | 0 | 28 | 72 | CS+TS |
| 28 | 0 | 23.33 | 76.67 | TS+M |
| 29 | 0 | 18 | 82 | TS+M |
| 30 | 0 | 13.33 | 86.67 | TS+M |
| 31 | 0 | 0 | 100 | M |
| 32 | 23 | 7 | 70 | CS |
| 33 | 20 | 8 | 72 | CS |
| 34 | 20 | 14 | 66 | CS |
| 35 | 20 | 20 | 60 | CS |
| 36 | 20 | 30 | 50 | CS |
| 37 | 20 | 40 | 40 | CS |
| 38 | 30.13 | 9.61 | 60.26 | CS+LM |
| 39 | 29.31 | 12.07 | 58.62 | CS+LM |
| 40 | 28.21 | 15.38 | 56.41 | CS+LM |
| 41 | 26.58 | 20.25 | 53.16 | CS+LM |
| 42 | 23.46 | 29.63 | 46.91 | CS+LM |
| 43 | 14.94 | 55.17 | 29.89 | CS |
| 44 | 11.11 | 66.67 | 22.22 | CS |
| 45 | 8.89 | 73.32 | 17.79 | CS |
| 46 | 6.57 | 80.28 | 13.14 | CS |
| 47 | 14.50 | 30.08 | 55.42 | CS |
| 48 | 9.09 | 40 | 50.91 | CS |
| 49 | 3.45 | 50.34 | 46.21 | CS |
| 50 | 24.22 | 10.14 | 65.63 | CS |
| 51 | 10 | 10 | 80 | M+TS+CS |
| 52 | 14 | 10 | 76 | TS+CS |
| 53 | 15 | 15 | 70 | TS+CS |
| 54 | 8 | 20 | 72 | TS+CS |
| 55 | 10 | 27 | 63 | TS+CS |
| 56 | 10 | 40 | 50 | CS |
| 57 | 10 | 60 | 30 | CS |
| 58 | 6 | 66.67 | 27.33 | CS |
| 59 | 16.67 | 66.67 | 16.67 | CS+LC2 |
| 60 | 20 | 66.67 | 13.33 | CS+LC2 |
| 61 | 23.33 | 66.23 | 10 | CS+LC2 |
| 62 | 30 | 66.67 | 3.33 | CS+LC2 |
| 63 | 12 | 80 | 8 | CS+LC2 |
| 64 | 6 | 90 | 4 | CS+LC2 |
| 65 | 30 | 40 | 30 | CS+LM+LC2 |
| 66 | 40 | 40 | 20 | LM+LC2 |
| 67 | 60 | 20 | 20 | L+LM+LC2 |
| 68 | 33.33 | 66.67 | 0 | LC2 |
| 69 | 30 | 20 | 50 | CS+LM |
| 70 | 22 | 20 | 58 | CS+LM |
| 71 | 27.5 | 12.5 | 60 | CS+LM |
